# Pseudo cardiac tamponade in the setting of excess pericardial fat

**DOI:** 10.1186/1476-7120-7-3

**Published:** 2009-01-22

**Authors:** Thang Nguyen, Kanwal Kumar, Andrew Francis, Jonathan R Walker, Michael Raabe, Shelley Zieroth, Davinder S Jassal

**Affiliations:** 1 Section of Cardiology, Department of Cardiac Sciences, University of Manitoba, Winnipeg, Manitoba, Canada; 2 Section of Cardiac Surgery, Department of Cardiac Sciences, University of Manitoba, Winnipeg, Manitoba, Canada; 3 Institute of Cardiovascular Sciences, St. Boniface Research Centre, University of Manitoba, Winnipeg, Manitoba, Canada; 4 Department of Radiology, University of Manitoba, Winnipeg, Manitoba, Canada

## Abstract

Cardiac tamponade is the phenomenon of hemodynamic compromise caused by a pericardial effusion. Following a myocardial infarction, the most common causes of pericardial fluid include early pericarditis, Dressler's syndrome, and hemopericardium secondary to a free wall rupture. On transthoracic echocardiography, pericardial fluid appears as an echo-free space in between the visceral and parietal layers of the pericardium. Pericardial fat has a similar appearance on echocardiography and it may be difficult to discern the two entities. We present a case of a post-MI patient demonstrating pseudo tamponade physiology in the setting of excessive pericardial fat.

## Background

Cardiac tamponade is the phenomenon of hemodynamic compromise caused by a pericardial effusion. There are a myriad of etiologies for pericardial effusions, including infectious, immune-mediated, and malignancy related causes. Following a myocardial infarction (MI), pericardial effusions can be caused by pericarditis, free wall rupture, or as a complication of percutaneous procedures [1]. The most efficient technique to diagnose cardiac tamponade is by transthoracic echocardiography (TTE). Pericardial effusions are detected as a lucent separation of parietal and visceral pericardium. Echocardiographic findings suggestive of tamponade physiology include early right ventricular (RV) diastolic collapse, right atrial (RA) systolic collapse, and respiratory variation greater than 40% across the mitral valve [2].

Normally, pericardial fat is located in the atrioventricular and interventricular grooves, along the major coronary arteries, around the atria and the apex of the left ventricle, as well as along the free wall of the right ventricle [3]. Using TTE, the mean epicardial fat thickness, measured at the free RV wall, is between 1 and 6 mm [4]. There has been some recent interest in the association between epicardial fat and atherosclerotic disease [5-7]. However, it is rare to have excess epicardial fat associated with tamponade physiology.

We present a case of pseudo cardiac tamponade in the setting of excess pericardial fat discovered after a non-ST-elevation myocardial infarction.

## Case presentation

A 66 year old woman presented to hospital with acute onset of shortness of breath. She was diagnosed as having a non-ST elevation myocardial infarction complicated by congestive heart failure at a community hospital. The patient was stabilized with medical therapy, including parenteral nitroglycerin and non-invasive pressure ventilation. Subsequently, she was referred to our tertiary care centre for a coronary angiogram. Her past medical history included type 2 diabetes mellitus, hypertension, smoking, and peripheral vascular disease.

Coronary angiogram revealed extensive coronary artery disease, including a 95% lesion of the left main coronary artery. There were no complications during the procedure. The left ventricular (LV) pressure was 90/45 mm Hg. While recovering from the angiogram, the patient became acutely dyspneic and tachycardic. Electrocardiogram revealed sinus tachycardia with no evidence of low voltages nor evidence of recurrent ischemia. Chest x-ray was consistent with pulmonary edema. Initial treatment included intravenous furosemide and nitroglycerin for acute congestive heart failure.

**Figure 1:**
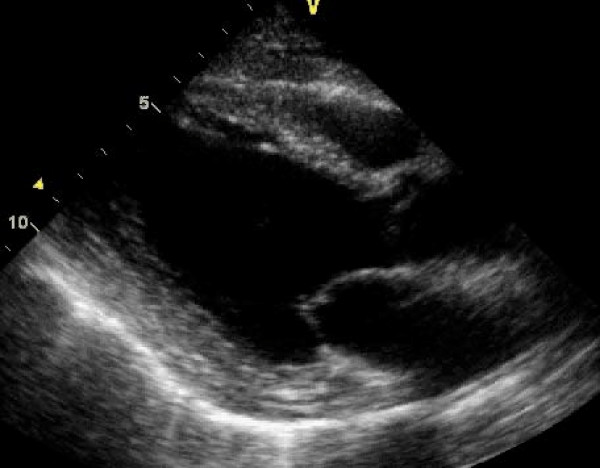
**A transthoracic echocardiogram parasternal long axis view demonstrating a mildly dilated left ventricle and global LV systolic dysfunction**.

**Figure 2:**
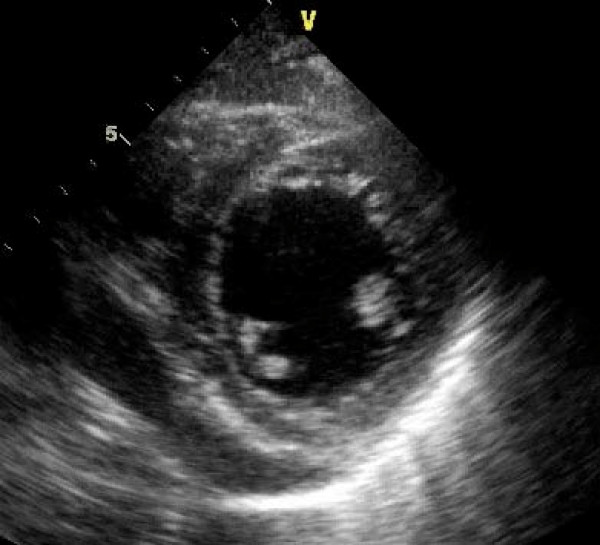
**A transthoracic echocardiogram parasternal short axis view demonstrating a mildly dilated left ventricle and global LV systolic dysfunction**.

**Figure 3:**
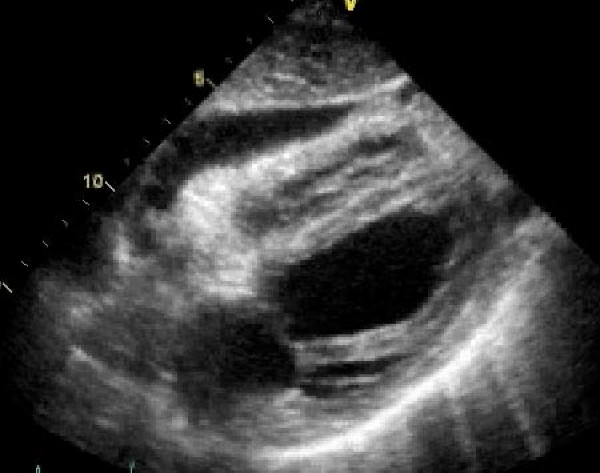
**A transthoracic echocardiogram 4 chamber view from subxiphoid approach representing a 25 mm echolucent region adjacent to the right ventricular free wall and right atrium, presumed to be due to a pericardial effusion**.

**Figure 4:**
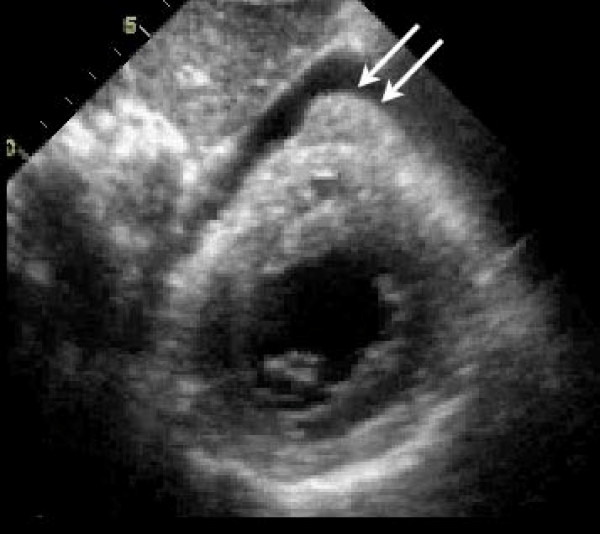
**A transthoracic echocardiographic short axis view from subxiphoid view representing echodense material attached to the right ventricular wall, presumed to be thrombus (arrows)**.

**Figure 5:**
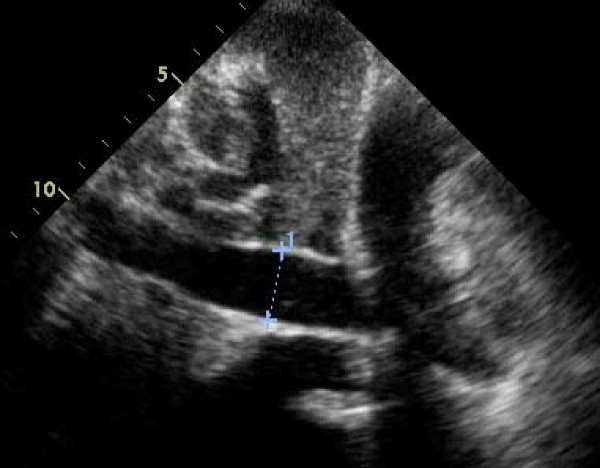
**A subxiphoid view of the dilated inferior vena cava greater than 2.0 cm**.

An urgent TTE was obtained to rule out complications related to ischemia or angiography. The left ventricle was mildly dilated with severe global systolic dysfunction and an ejection fraction of 20–25% (Figures 1 and 2, see Additional files 1 and 2). There was mild mitral regurgitation with a pulmonary artery systolic pressure of 30 mm Hg. No mechanical complications due to myocardial ischemia were noted. A moderate sized pericardial effusion, measuring 25 mm in diameter was present, particularly along the RV free wall (Figure 3, see Additional file 3). Echodense material attached to the RV was also observed, and was presumed to be thrombus (Figure 4, see Additional file 4). The inferior vena cava was dilated and did not collapse with inspiration (Figure 5). Right atrial systolic collapse and mild RV inversion was suggestive of cardiac tamponade physiology.

The patient was taken to the operating room for emergent coronary artery bypass grafting and management of the presumed hemodynamic significant pericardial effusion. While dissecting for visualization of the coronary arteries, a large amount of epicardial fat was noted covering the heart. There was no evidence of pericardial fluid or thrombus found during the surgery. Following successful revascularization and removal of the epicardial fat, the 'tamponade' physiology resolved. Post-operatively, the patient's admission was uneventful, with no further symptoms of chest pain or shortness of breath. She fully recovered and was discharged within four days.

## Discussion

Although pericardial fat with effusion has been previously documented, we are unaware of previous case reports demonstrating tamponade physiology in the setting of excess pericardial fat. There has been one case report of pericardial fat mimicking effusion in a hypotensive patient, which led to a pericardiotomy, however echocardiographic examination of that patient did not reveal cardiac tamponade physiology [8]. The echocardiographic evidence of tamponade physiology secondary to 'presumed' pericardial effusion in the current case likely represented acute ischemia from the severe left main disease and incidental pericardial fat, which resolved following successful revascularization.

In the post-MI setting, there are several potential etiologies for pericardial effusions. Early post-MI infarction pericarditis is a well recognized entity. It occurs roughly 1 to 2 days after the infarction, and is usually associated with large anterior ST-elevation myocardial infarctions [9]. Pericardial effusions occur in 28% of patients shortly after a MI and rarely develop tamponade on its own [9,10]. Dressler's syndrome, also known as post MI syndrome, is believed to be an autoimmune reaction of myocytes causing polymyoserositis. This entity usually appears one week to three months post-MI. Since the era of early reperfusion with thrombolytics, the incidence of Dressler's syndrome is now quite rare [11]. There have been several case reports of tamponade physiology secondary to Dressler's syndrome [12,13]. Another potential etiology is a free wall rupture after an ST-elevation MI, which can create a large hemopericardium and subsequent cardiac tamponade. In patients with cardiogenic shock post-MI, 2.7% of the patients had free wall rupture as the cause for their shock [14]. Lastly, patients undergoing percutaneous coronary intervention (PCI) can have an iatrogenic cause for pericardial effusions. In general, coronary artery perforation with pericardial effusion development occurs at a rate of 0.1–3.0% of PCI procedures, of which less than 0.5% require pericardial drainage [15].

Pericardial fat appears as an echo free space mimicking the echocardiographic appearance of pericardial effusions. Differentiating the two entities can be difficult, not infrequently leading to alternative imaging modalities or surgery for confirmation [16,17]. Since pericardial fluid tends to collect in the dependent areas, echo free space predominantly in the posterior portion suggests fluid. Meanwhile, echo free space confined to the anterior portion is more consistent with pericardial fat. However, large effusions can collect anteriorly and autopsy results have demonstrated fat posteriorly [16]. In order to obtain an accurate diagnosis, alternative imaging modalities including cardiac magnetic resonance imaging (CMR) or computed tomography (CT) can be utilized. For CMR, spin echo sequences with short repetition time and echo time give fat a high intensity signal and easily differentiates fat from fluid or soft tissue [17]. On CT scans, pericardial fat can be distinguished by its density or Hounsfield units. Pericardial effusions would attenuate x-rays at a similar level as water. Adipose tissue and its higher density would demonstrate greater attenuation of x-rays, and thus a higher value of Hounsfield units [9].

## Conclusion

Although rare, excessive pericardial fat in the setting of active ischemia, should be considered in the differential diagnosis of any patient presenting with a "pericardial effusion" and pseudo tamponade on TTE following percutaneous coronary angiography.

## Competing interests

The authors declare that they have no competing interests.

## Authors' contributions

TN, AF, JW and DJ wrote the manuscript. KK, MR, SZ and DJ were involved in the patient's clinical care. All authors read and approved the final manuscript.

## Consent

Written informed consent was obtained from the patient for publication of this case report and any accompanying images. A copy of the written consent is available for review by the Editor-in-Chief of this journal.

## Supplementary Material

Additional file 1 **Movie 1**. A transthoracic echocardiogram parasternal long axis view demonstrating a mildly dilated left ventricle and global LV systolic dysfunction. Click here for file

Additional file 2 **Movie 2**. A transthoracic echocardiogram parasternal short axis view demonstrating a mildly dilated left ventricle and global LV systolic dysfunction. Click here for file

Additional file 3 **Movie 3**. A transthoracic echocardiogram 4 chamber view from subxiphoid approach representing a 25 mm echolucent region adjacent to the right ventricular free wall and right atrium, presumed to be due to a pericardial effusion. There was echocardiographic evidence suggestive of tamponade physiology with right atrial systolic collapse and right ventricular early diastolic collapse. Click here for file

Additional file 4 **Movie 4**. A transthoracic echocardiographic short axis view from subxiphoid view representing echodense material attached to the right ventricular wall, presumed to be thrombus. Click here for file
